# Cognitive neuroscience perspective on memory: overview and summary

**DOI:** 10.3389/fnhum.2023.1217093

**Published:** 2023-07-26

**Authors:** Sruthi Sridhar, Abdulrahman Khamaj, Manish Kumar Asthana

**Affiliations:** ^1^Department of Psychology, Mount Allison University, Sackville, NB, Canada; ^2^Department of Industrial Engineering, College of Engineering, Jazan University, Jazan, Saudi Arabia; ^3^Department of Humanities and Social Sciences, Indian Institute of Technology Roorkee, Roorkee, India; ^4^Department of Design, Indian Institute of Technology Roorkee, Roorkee, India

**Keywords:** memory, cellular consolidation, cognitive neuroscience, hippocampus, sleep

## Abstract

This paper explores memory from a cognitive neuroscience perspective and examines associated neural mechanisms. It examines the different types of memory: working, declarative, and non-declarative, and the brain regions involved in each type. The paper highlights the role of different brain regions, such as the prefrontal cortex in working memory and the hippocampus in declarative memory. The paper also examines the mechanisms that underlie the formation and consolidation of memory, including the importance of sleep in the consolidation of memory and the role of the hippocampus in linking new memories to existing cognitive schemata. The paper highlights two types of memory consolidation processes: cellular consolidation and system consolidation. Cellular consolidation is the process of stabilizing information by strengthening synaptic connections. System consolidation models suggest that memories are initially stored in the hippocampus and are gradually consolidated into the neocortex over time. The consolidation process involves a hippocampal-neocortical binding process incorporating newly acquired information into existing cognitive schemata. The paper highlights the role of the medial temporal lobe and its involvement in autobiographical memory. Further, the paper discusses the relationship between episodic and semantic memory and the role of the hippocampus. Finally, the paper underscores the need for further research into the neurobiological mechanisms underlying non-declarative memory, particularly conditioning. Overall, the paper provides a comprehensive overview from a cognitive neuroscience perspective of the different processes involved in memory consolidation of different types of memory.

## Introduction

Memory is an essential cognitive function that permits individuals to acquire, retain, and recover data that defines a person’s identity (Zlotnik and Vansintjan, 2019). Memory is a multifaceted cognitive process that involves different stages: encoding, consolidation, recovery, and reconsolidation. Encoding involves acquiring and processing information that is transformed into a neuronal representation suitable for storage (Liu et al., 2021; Panzeri et al., 2023). The information can be acquired through various channels, such as visual, auditory, olfactory, or tactile inputs. The acquired sensory stimuli are converted into a format the brain can process and retain. Different factors such as attention, emotional significance, and repetition can influence the encoding process and determine the strength and durability of the resulting memory (Squire et al., 2004; Lee et al., 2016; Serences, 2016).

Consolidation includes the stabilization and integration of memory into long-term storage to increase resistance to interference and decay (Goedert and Willingham, 2002). This process creates enduring structural modification in the brain and thereby has consequential effects on the function by reorganizing and strengthening neural connections. Diverse sources like sleep and stress and the release of neurotransmitters can influence memory consolidation. Many researchers have noted the importance of sleep due to its critical role in enabling a smooth transition of information from transient repositories into more stable engrams (memory traces) (McGaugh, 2000; Clawson et al., 2021; Rakowska et al., 2022).

Retrieval involves accessing, selecting, and reactivating or reconstructing the stored memory to allow conscious access to previously encoded information (Dudai, 2002). Retrieving memories depends on activating relevant neural pathways while reconstructing encoded information. Factors like contextual or retrieval cues and familiarity with the material can affect this process. Forgetting becomes a possibility if there are inadequate triggers for associated memory traces to activate upon recall. Luckily, mnemonic strategies and retrieval practice offer effective tools to enhance recovery rates and benefit overall memory performance (Roediger and Butler, 2011).

Previous research implied that once a memory has been consolidated, it becomes permanent (McGaugh, 2000; Robins, 2020). However, recent studies have found an additional phase called “reconsolidation,” during which stored memories, when reactivated, enter a fragile or liable state and become susceptible to modification or update (Schiller et al., 2009; Asthana et al., 2015). The process highlights the notion that memory is not static but a dynamic system influenced by subsequent encounters. The concept of reconsolidation has much significance in memory modification therapies and interventions, as it offers a promising opportunity to target maladaptive or traumatic memories for modification specifically. However, more thorough investigations are needed to gain insight into the mechanisms and concrete implications of employing memory reconsolidation within therapeutic settings (Bellfy and Kwapis, 2020).

The concept of memory is not reducible to a single unitary phenomenon; instead, evidence suggests that it can be subdivided into several distinct but interrelated constituent processes and systems (Richter-Levin and Akirav, 2003). There are three major types of human memory: working memory, declarative memory (explicit), and non-declarative memory (implicit). All these types of memories involve different neural systems in the brain. Working memory is a unique transient active store capable of manipulating information essential for many complex cognitive operations, including language processing, reasoning, and judgment (Atkinson and Shiffrin, 1968; Baddeley and Logie, 1999; Funahashi, 2017; Quentin et al., 2019). Previous models suggest the existence of three components that make up the working memory (Baddeley and Hitch, 1974; Baddeley, 1986). One master component, the central executive, controls the two dependent components, the phonological loop (speech perception and language comprehension) and the visuospatial sketchpad (visual images and spatial impressions processing). Some models mention a third component known as the episodic buffer. It is theorized that the episodic buffer serves as an intermediary between perception, long-term memory, and two components of working memory (the phonological loop and visuospatial sketchpad) by storing integrated episodes or chunks from both sources (Baddeley, 2000). Declarative memory (explicit memory) can be recalled consciously, including facts and events that took place in one’s life or information learned from books. It encompasses memories of both autobiographical experiences and memories associated with general knowledge. It is usually associated with the hippocampus–medial temporal lobe system (Thompson and Kim, 1996; Ober, 2014). Non-declarative memory (implicit memory) refers to unconscious forms of learning such as skills, habits, and priming effects; this type of implicit learning does not involve conscious recollection but can include motor skill tasks that often require no thought prior to execution nor later recall upon completion. This type of memory usually involves the amygdala and other systems (Thompson and Kim, 1996; Ober, 2014).

## Working memory

Working memory is primarily associated with the prefrontal and posterior parietal cortex (Sarnthein et al., 1998; Todd and Marois, 2005). Working memory is not localized to a single brain region, and research suggests that it is an emergent property arising from functional interactions between the prefrontal cortex (PFC) and the rest of the brain (D’Esposito, 2007). Neuroimaging studies have explored the neural basis for the three components proposed by Baddeley and Hitch (1974), the Central executive, the phonological loop, and the visuospatial sketch pad; there is evidence for the existence of a fourth component called the episodic buffer (Baddeley, 2000).

The central executive plays a significant role in working memory by acting as the control center (Shallice, 2002). It facilitates critical functions like attention allocation and coordination between the phonological loop and the visuospatial sketchpad (Yu et al., 2023). Recent findings have illuminated the dual-functional network regulation, the cingulo-opercular network (CON) and the frontoparietal network (FPN), that underpins the central executive system (Yu et al., 2023). The CON comprises the dorsal anterior cingulate cortex (dACC) and anterior insula (AI). In contrast, the FPN encompasses various regions, such as the dorsolateral prefrontal cortex (DLPFC) and frontal eye field (FEF), along with the intraparietal sulcus (IPS) (Yu et al., 2023). Neuroimaging research has found evidence that elucidates the neural underpinnings of the executive attention control system to the dorsolateral prefrontal cortex (DLPFC) and the anterior cingulate cortex (ACC) (Jung et al., 2022). The activation patterns indicate that the CON may have a broader top-down control function across the working memory process. At the same time, the FPN could be more heavily implicated in momentary control or processing at the trial level (Yu et al., 2023). Evidence suggests that the central executive interacts with the phonological loop and visuospatial sketchpad to support working memory processes (Baddeley, 2003; Buchsbaum, 2010; Menon and D’Esposito, 2021). The function, localization, and neural basis of this interaction are thought to involve the activation of specific brain regions associated with each component of working memory, as discussed in detail below.

The phonological loop is divided into two components: a storage system that maintains information (a few seconds) and a component involving subvocal rehearsal—which maintains and refreshes information in the working memory. Neuroanatomically, the phonological loop is represented in the Brodmann area (BA) 40 in the parietal cortex and the rehearsal components in BA 44 and 6, both situated in the frontal cortex (Osaka et al., 2007). The left inferior frontal gyrus (Broca’s area) and the left posterior superior temporal gyrus (Wernicke’s area) has been proposed to play a critical role in supporting phonological and verbal working memory tasks, specifically the subvocal rehearsal system of the articulatory loop (Paulesu et al., 1993; Buchsbaum et al., 2001; Perrachione et al., 2017). The phonological store in verbal short-term memory has been localized at the left supramarginal gyrus (Graves et al., 2008; Perrachione et al., 2017).

Studies utilizing neuroimaging techniques have consistently yielded results indicating notable activation in these brain regions during phonological activities like recalling non-words and maintaining verbal information in memory (Awh et al., 1996; Graves et al., 2008). During tasks that require phonological rehearsal, there was an increase in activation in the left inferior frontal gyrus (Paulesu et al., 1993). Researchers have noted an increase in activity within the superior temporal gyrus-which plays a significant role in auditory processing-in individuals performing tasks that necessitate verbal information maintenance and manipulation (Smith et al., 1998; Chein et al., 2003).

Additionally, lesion studies have provided further confirmation regarding the importance of these regions. These investigations have revealed that impairment in performing phonological working memory tasks can transpire following damage inflicted upon the left hemisphere, particularly on perisylvian language areas (Koenigs et al., 2011). It is common for individuals with lesions affecting regions associated with the phonological loop, such as the left inferior frontal gyrus and superior temporal gyrus, to have difficulty performing verbal working memory tasks. Clinical cases involving patients diagnosed with aphasia and specific language impairments have highlighted challenges related to retaining and manipulating auditory information. For example, those who sustain damage specifically within their left inferior frontal gyrus often struggle with tasks involving phonological rehearsal and verbal working memory activities, and therefore, they tend to perform poorly in tasks that require manipulation or repetition of verbal stimuli (Saffran, 1997; Caplan and Waters, 2005).

The visuospatial sketchpad is engaged in the temporary retention and manipulation of visuospatial facts, including mental pictures, spatial associations, and object placements (Miyake et al., 2001). The visuospatial sketchpad is localized to the right hemisphere, including the occipital lobe, parietal and frontal areas (Osaka et al., 2007). Ren et al. (2019) identified the localization of the visuospatial sketchpad, and these areas were the right infero-lateral prefrontal cortex, lateral pre-motor cortices, right inferior parietal cortex, and the dorsolateral occipital cortices (Burbaud et al., 1999; Salvato et al., 2021). Moreover, the posterior parietal cortex and the intraparietal sulcus have been implicated in spatial working memory (Xu and Chun, 2006). Additionally, some evidence is available for an increase in brain regions associated with the visuospatial sketchpad during tasks involving mental imagery and spatial processing. Neuroimaging studies have revealed increased neural activation in some regions of the parietal cortex, mainly the superior and posterior parietal cortex, while performing mental rotation tasks (Cohen et al., 1996; Kosslyn et al., 1997). However, further research is needed to better understand the visuospatial working memory and its integration with other cognitive processes (Baddeley, 2003). Lesions to the regions involving the visuospatial sketchpad can have detrimental effects on visuospatial working memory tasks. Individuals with lesions to the posterior parietal cortex may exhibit deficits in mental rotation tasks and may be unable to mentally manipulate the visuospatial representation (Buiatti et al., 2011). Moreover, studies concerning lesions have shown that damage to the parietal cortex can result in short-term deficits in visuospatial memory (Shafritz et al., 2002). Damage to the occipital cortex can lead to performance impairments in tasks that require the generation and manipulation of mental visual images (Moro et al., 2008).

The fourth component of the working memory, termed episodic buffer, was proposed by Baddeley (2000). The episodic buffer is a multidimensional but essentially passive store that can hold a limited number of chunks, store bound features, and make them available to conscious awareness (Baddeley et al., 2010; Hitch et al., 2019). Although research has suggested that episodic buffer is localized to the hippocampus (Berlingeri et al., 2008) or the inferior lateral parietal cortex, it is thought to be not dependent on a single anatomical structure but instead can be influenced by the subsystems of working memory, long term memory, and even through perception (Vilberg and Rugg, 2008; Baddeley et al., 2010). The episodic buffer provides a crucial link between the attentional central executive and the multidimensional information necessary for the operation of working memory (Baddeley et al., 2011; Gelastopoulos et al., 2019).

The interdependence of the working memory modules, namely the phonological loop and visuospatial sketchpad, co-relates with other cognitive processes, for instance, spatial cognition and attention allocation (Repovs and Baddeley, 2006). It has been found that the prefrontal cortex (PFC) and posterior parietal cortex (PPC) have a crucial role in several aspects of spatial cognition, such as the maintenance of spatially oriented attention and motor intentions (Jerde and Curtis, 2013). The study by Sellers et al. (2016) and the review by Ikkai and Curtis (2011) posits that other brain areas could use the activity in PFC and PPC as a guide and manifest outputs to guide attention allocation, spatial memory, and motor planning. Moreover, research indicates that verbal information elicits an activation response in the left ventrolateral prefrontal cortex (VLPFC) when retained in the phonological loop, while visuospatial information is represented by a corresponding level of activity within the right homolog region (Narayanan et al., 2005; Wolf et al., 2006; Emch et al., 2019). Specifically, the study by Yang et al. (2022) investigated the roles of two regions in the brain, the right inferior frontal gyrus (rIFG) and the right supra-marginal gyrus (rSMG), as they relate to spatial congruency in visual working memory tasks. A change detection task with online repetitive transcranial magnetic stimulation applied concurrently at both locations during high visual WM load conditions determined that rIFG is involved in actively repositioning the location of objects. At the same time, rSMG is engaged in passive perception of the stability of the location of objects.

Recent academic studies have found evidence to support the development of a new working memory model known as the state-based model (D’Esposito and Postle, 2015). This theoretical model proposes that the allocation of attention toward internal representations permits short-term retention within working memory (Ghaleh et al., 2019). The state-based model consists of two main categories: activated LTM models and sensorimotor recruitment models; the former largely focuses upon symbolic stimuli categorized under semantic aspects, while the latter has typically been applied to more perceptual tasks in experiments. This framework posits that prioritization through regulating cognitive processes provides insight into various characteristics across different activity types, including capacity limitations, proactive interference, etcetera (D’Esposito and Postle, 2015). For example, the paper by Ghaleh et al. (2019) provides evidence for two separate mechanisms involved in maintenance of auditory information in verbal working memory: an articulatory rehearsal mechanism that relies more heavily on left sensorimotor areas and a non-articulatory maintenance mechanism that critically relies on left superior temporal gyrus (STG). These findings support the state-based model’s proposal that attentional allocation is necessary for short-term retention in working memory.

State-based models were found to be consistent with the suggested storage mechanism as they do not require representation transfer from one dedicated buffer type; research has demonstrated that any population of neurons and synapses may serve as such buffers (Maass and Markram, 2002; Postle, 2006; Avraham et al., 2017). The review by D’Esposito and Postle (2015) examined the evidence to determine whether a persistent neural activity, synaptic mechanisms, or a combination thereof support representations maintained during working memory. Numerous neural mechanisms have been hypothesized to support the short-term retention of information in working memory and likely operate in parallel (Sreenivasan et al., 2014; Kamiński and Rutishauser, 2019).

Persistent neural activity is the neural mechanism by which information is temporarily maintained (Ikkai and Curtis, 2011; Panzeri et al., 2023). Recent review by Curtis and Sprague (2021) has focused on the notion that persistent neural activity is a fundamental mechanism for memory storage and have provided two main arcs of explanation. The first arc, mainly underpinned by empirical evidence from prefrontal cortex (PFC) neurophysiology experiments and computational models, posits that PFC neurons exhibit sustained firing during working memory tasks, enabling them to store representations in their active state (Thuault et al., 2013). Intrinsic persistent firing in layer V neurons in the medial PFC has been shown to be regulated by HCN1 channels, which contribute to the executive function of the PFC during working memory episodes (Thuault et al., 2013). Additionally, research has also found that persistent neural firing could possibly interact with theta periodic activity to sustain each other in the medial temporal, prefrontal, and parietal regions (Düzel et al., 2010; Boran et al., 2019). The second arc involves advanced neuroimaging approaches which have, more recently, enabled researchers to decode content stored within working memories across distributed regions of the brain, including parts of the early visual cortex–thus extending this framework beyond just isolated cortical areas such as the PFC. There is evidence that suggests simple, stable, persistent activity among neurons in stimulus-selective populations may be a crucial mechanism for sustaining WM representations (Mackey et al., 2016; Kamiński et al., 2017; Curtis and Sprague, 2021).

Badre (2008) discussed the functional organization of the PFC. The paper hypothesized that the rostro-caudal gradient of a function in PFC supported a control hierarchy, whereas posterior to anterior PFC mediated progressively abstract, higher-order controls (Badre, 2008). However, this outlook proposed by Badre (2008) became outdated; the paper by Badre and Nee (2018) presented an updated look at the literature on hierarchical control. This paper supports neither a unitary model of lateral frontal function nor a unidimensional abstraction gradient. Instead, separate frontal networks interact via local and global hierarchical structures to support diverse task demands. This updated perspective is supported by recent studies on the hierarchical organization of representations within the lateral prefrontal cortex (LPFC) and the progressively rostral areas of the LPFC that process/represent increasingly abstract information, facilitating efficient and flexible cognition (Thomas Yeo et al., 2011; Nee and D’Esposito, 2016). This structure allows the brain to access increasingly abstract action representations as required (Nee and D’Esposito, 2016). It is supported by fMRI studies showing an anterior-to-posterior activation movement when tasks become more complex. Anatomical connectivity between areas also supports this theory, such as Area 10, which has projections back down to Area 6 but not vice versa.

Finally, studies confirm that different regions serve different roles along a hierarchy leading toward goal-directed behavior (Badre and Nee, 2018). The paper by Postle (2015) exhibits evidence of activity in the prefrontal cortex that reflects the maintenance of high-level representations, which act as top-down signals, and steer the circulation of neural pathways across brain networks. The PFC is a source of top-down signals that influence processing in the posterior and subcortical regions (Braver et al., 2008; Friedman and Robbins, 2022). These signals either enhance task-relevant information or suppress irrelevant stimuli, allowing for efficient yet effective search (D’Esposito, 2007; D’Esposito and Postle, 2015; Kerzel and Burra, 2020). The study by Ratcliffe et al. (2022) provides evidence of the dynamic interplay between executive control mechanisms in the frontal cortex and stimulus representations held in posterior regions for working memory tasks. Moreover, the review by Herry and Johansen (2014) discusses the neural mechanisms behind actively maintaining task-relevant information in order for a person to carry out tasks and goals effectively. This review of data and research suggests that working memory is a multi-component system allowing for both the storage and processing of temporarily active representations. Neural activity throughout the brain can be differentially enhanced or suppressed based on context through top-down signals emanating from integrative areas such as PFC, parietal cortex, or hippocampus to actively maintain task-relevant information when it is not present in the environment (Herry and Johansen, 2014; Kerzel and Burra, 2020).

In addition, Yu et al. (2022) examined how brain regions from the ventral stream pathway to the prefrontal cortex were activated during working memory (WM) gate opening and closing. They defined gate opening as the switch from maintenance to updating and gate closing as the switch from updating to maintenance. The data suggested that cognitive branching increases during the WM gating process, thus correlating the gating process and an information approach to the PFC function. The temporal cortices, lingual gyrus (BA19), superior frontal gyri including frontopolar cortices, and middle and inferior parietal regions are involved in processes of estimating whether a response option available will be helpful for each case. During gate closing, on the other hand, medial and superior frontal regions, which have been associated with conflict monitoring, come into play, as well as orbitofrontal and dorsolateral prefrontal processing at later times when decreasing activity resembling stopping or downregulating cognitive branching has occurred, confirming earlier theories about these areas being essential for estimation of usefulness already stored within long-term memories (Yu et al., 2022).

## Declarative and non-declarative memory

The distinctions between declarative and non-declarative memory are often based on the anatomical features of medial temporal lobe regions, specifically those involving the hippocampus (Squire and Zola, 1996; Squire and Wixted, 2011). In the investigation of systems implicated in the process of learning and memory formation, it has been posited that the participation of the hippocampus is essential for the acquisition of declarative memories (Eichenbaum and Cohen, 2014). In contrast, a comparatively reduced level of hippocampal involvement may suffice for non-declarative memories (Squire and Zola, 1996; Williams, 2020).

Declarative memory (explicit) pertains to knowledge about facts and events. This type of information can be consciously retrieved with effort or spontaneously recollected without conscious intention (Dew and Cabeza, 2011). There are two types of declarative memory: Episodic and Semantic. Episodic memory is associated with the recollection of personal experiences. It involves detailed information about events that happened in one’s life. Semantic memory refers to knowledge stored in the brain as facts, concepts, ideas, and objects; this includes language-related information like meanings of words and mathematical symbol values along with general world knowledge (e.g., capitals of countries) (Binder and Desai, 2011). The difference between episodic and semantic memory is that when one retrieves episodic memory, the experience is known as “remembering”; when one retrieves information from semantic memory, the experience is known as “knowing” (Tulving, 1985; Dew and Cabeza, 2011). The hippocampus, medial temporal lobe, and the areas in the diencephalon are implicated in declarative memory (Richter-Levin and Akirav, 2003; Derner et al., 2020). The ventral parietal cortex (VPC) is involved in declarative memory processes, specifically episodic memory retrieval (Henson et al., 1999; Davis et al., 2018). The evidence suggests that VPC and hippocampus is involved in the retrieval of contextual details, such as the location and timing of the event, and the information is critical for the formation of episodic memory (Daselaar, 2009; Hutchinson et al., 2009; Wiltgen et al., 2010). The prefrontal cortex (PFC) is involved in the encoding (medial PFC) and retrieval (lateral PFC) of declarative memories, specifically in the integration of information across different sensory modalities (Blumenfeld and Ranganath, 2007; Li et al., 2010). Research also suggests that the amygdala may modulate other brain regions involved with memory processing, thus, contributing to an enhanced recall of negative or positive experiences (Hamann, 2001; Ritchey et al., 2008; Sendi et al., 2020). Maintenance of the integrity of hippocampal circuitry is essential for ensuring that episodic memory, along with spatial and temporal context information, can be retained in short-term or long-term working memory beyond 15 min (Ito et al., 2003; Rasch and Born, 2013). Moreover, studies have suggested that the amygdala plays a vital role in encoding and retrieving explicit memories, particularly those related to emotionally charged stimuli which are supported by evidence of correlations between hippocampal activity and amygdala modulation during memory formation (Richter-Levin and Akirav, 2003; Qasim et al., 2023).

Current findings in neuroimaging studies assert that a vast array of interconnected brain regions support semantic memory (Binder and Desai, 2011). This network merges information sourced from multiple senses alongside different cognitive faculties necessary for generating abstract supramodal views on various topics stored within our consciousness. Modality-specific sensory, motor, and emotional system within these brain regions serve specialized tasks like language comprehension, while larger areas of the brain, such as the inferior parietal lobe and most of the temporal lobe, participate in more generalized interpretation tasks (Binder and Desai, 2011; Kuhnke et al., 2020). These regions lie at convergences of multiple perceptual processing streams, enabling increasingly abstract, supramodal representations of perceptual experience that support a variety of conceptual functions, including object recognition, social cognition, language, and the remarkable human capacity to remember the past and imagine the future (Binder and Desai, 2011; Binney et al., 2016). The following section will discuss the processes underlying memory consolidation and storage within declarative memory.

Non-declarative (implicit) memories refer to unconscious learning through experience, such as habits and skills formed from practice rather than memorizing facts; these are typically acquired slowly and automatically in response to sensory input associated with reward structures or prior exposure within our daily lives (Kesner, 2017). Non-declarative memory is a collection of different phenomena with different neural substrates rather than a single coherent system (Camina and Güell, 2017). It operates by similar principles, depending on local changes to a circumscribed brain region, and the representation of these changes is unavailable to awareness (Reber, 2008). Non-declarative memory encompasses a heterogenous collection of abilities, such as associative learning, skills, and habits (procedural memory), priming, and non-associative learning (Squire and Zola, 1996; Camina and Güell, 2017). Studies have concluded that procedural memory for motor skills depends upon activity in diverse set areas such as the motor cortex, striatum, limbic system, and cerebellum; similarly, perceptual skill learning is thought to be associated with sensory cortical activation (Karni et al., 1998; Mayes, 2002). Research suggests that mutual connections between brain regions that are active together recruit special cells called associative memory cells (Wang et al., 2016; Wang and Cui, 2018). These cells help integrate, store, and remember related information. When activated, these cells trigger the recall of memories, leading to behaviors and emotional responses. This suggests that co-activated brain regions with these mutual connections are where associative memories are formed (Wang et al., 2016; Wang and Cui, 2018). Additionally, observational data reveals that priming mechanisms within distinct networks, such as the “repetition suppression” effect observed in visual cortical areas associated with sensory processing and in the prefrontal cortex for semantic priming, are believed to be responsible for certain forms of conditioning and implicit knowledge transfer experiences exhibited by individuals throughout their daily lives (Reber, 2008; Wig et al., 2009; Camina and Güell, 2017). However, further research is needed to better understand the mechanisms of consolidation in non-declarative memory (Camina and Güell, 2017).

The process of transforming memory into stable, long-lasting from a temporary, labile memory is known as memory consolidation (McGaugh, 2000). Memory formation is based on the change in synaptic connections of neurons representing the memory. Encoding causes synaptic Long-Term potentiation (LTP) or Long-Term depression (LTD) and induces two consolidation processes. The first is synaptic or cellular consolidation which involves remodeling synapses to produce enduring changes. *Cellular consolidation* is a short-term process that involves stabilizing the neural trace shortly after learning via structural brain changes in the hippocampus (Lynch, 2004). The second is system consolidation, which builds on synaptic consolidation where reverberating activity leads to redistribution for long-term storage (Mednick et al., 2011; Squire et al., 2015). *System consolidation* is a long-term process during which memories are gradually transferred to and integrated with cortical neurons, thus promoting their stability over time. In this way, memories are rendered less susceptible to forgetting. Hebb postulated that when two neurons are repeatedly activated simultaneously, they become more likely to exhibit a coordinated firing pattern of activity in the future (Langille, 2019). This proposed enduring change in synchronized neuronal activation was consequently termed cellular consolidation (Bermudez-Rattoni, 2010).

The following sections of this paper incorporate a more comprehensive investigation into various essential procedures connected with memory consolidation- namely: long-term potentiation (LTP), long-term depression (LTD), system consolidation, and cellular consolidation. Although these mechanisms have been presented briefly before this paragraph, the paper aims to offer greater insight into each process’s function within the individual capacity and their collective contribution toward memory consolidation.

## Synaptic plasticity mechanisms implicated in memory stabilization

Long-Term Potentiation (LTP) and Long-Term Depression (LTP) are mechanisms that have been implicated in memory stabilization. LTP is an increase in synaptic strength, whereas LTD is a decrease in synaptic strength (Ivanco, 2015; Abraham et al., 2019).

Long-Term Potentiation (LTP) is a phenomenon wherein synaptic strength increases persistently due to brief exposures to high-frequency stimulation (Lynch, 2004). Studies of Long-Term Potentiation (LTP) have led to an understanding of the mechanisms behind synaptic strengthening phenomena and have provided a basis for explaining how and why strong connections between neurons form over time in response to stimuli.

The NMDA receptor-dependent LTP is the most commonly described LTP (Bliss and Collingridge, 1993; Luscher and Malenka, 2012). In this type of LTP, when there is high-frequency stimulation, the presynaptic neuron releases glutamate, an excitatory neurotransmitter. Glutamate binds to the AMPA receptor on the postsynaptic neuron, which causes the neuron to fire while opening the NMDA receptor channel. The opening of an NMDA channel elicits a calcium ion influx into the postsynaptic neuron, thus initiating a series of phosphorylation events as part of the ensuing molecular cascade. Autonomously phosphorylated CaMKII and PKC, both actively functional through such a process, have been demonstrated to increase the conductance of pre-existing AMPA receptors in synaptic networks. Additionally, this has been shown to stimulate the introduction of additional AMPA receptors into synapses (Malenka and Nicoll, 1999; Lynch, 2004; Luscher and Malenka, 2012; Bailey et al., 2015).

There are two phases of LTP: the early phase and the late phase. It has been established that the early phase LTP (E-LTP) does not require RNA or protein synthesis; therefore, its synaptic strength will dissipate in minutes if late LTP does not stabilize it. On the contrary, late-phase LTP (L-LTP) can sustain itself over a more extended period, from several hours to multiple days, with gene transcription and protein synthesis in the postsynaptic cell (Frey and Morris, 1998; Orsini and Maren, 2012). The strength of presynaptic tetanic stimulation has been demonstrated to be a necessary condition for the activation of processes leading to late LTP (Luscher and Malenka, 2012; Bailey et al., 2015). This finding is supported by research examining synaptic plasticity, notably Eric Kandel’s discovery that CREB–a transcription factor–among other cytoplasmic and nuclear molecules, are vital components in mediating molecular changes culminating in protein synthesis during this process (Kaleem et al., 2011; Kandel et al., 2014). Further studies have shown how these shifts ultimately lead to AMPA receptor stabilization at post-synapses facilitating long-term potentiation within neurons (Luscher and Malenka, 2012; Bailey et al., 2015).

The “synaptic tagging and capture hypothesis” explains how a weak event of tetanization at synapse A can transform to late-LTP if followed shortly by the strong tetanization of a different, nearby synapse on the same neuron (Frey and Morris, 1998; Redondo and Morris, 2011; Okuda et al., 2020; Park et al., 2021). During this process, critical plasticity-related proteins (PRPs) are synthesized, which stabilize their own “tag” and that from the weaker synaptic activity (Moncada et al., 2015). Recent evidence suggests that calcium-permeable AMPA receptors (CP-AMPARs) are involved in this form of heterosynaptic metaplasticity (Park et al., 2018). The authors propose that the synaptic activation of CP-AMPARs triggers the synthesis of PRPs, which are then engaged by the weak induction protocol to facilitate LTP on the independent input. The paper also suggests that CP-AMPARs are required during the induction of LTP by the weak input for the full heterosynaptic metaplastic effect to be observed (Park et al., 2021). Additionally, it has been further established that catecholamines such as dopamine plays an integral part in memory persistence by inducing PRP synthesis (Redondo and Morris, 2011; Vishnoi et al., 2018). Studies have found that dopamine release in the hippocampus can enhance LTP and improve memory consolidation (Lisman and Grace, 2005; Speranza et al., 2021).

Investigations into neuronal plasticity have indicated that synaptic strength alterations associated with certain forms of learning and memory may be analogous to those underlying Long-Term Potentiation (LTP). Research has corroborated this notion, demonstrating a correlation between these two phenomena (Lynch, 2004). The three essential properties of Long-Term Potentiation (LTP) that have been identified are associativity, synapse specificity, and cooperativity (Kandel and Mack, 2013). These characteristics provide empirical evidence for the potential role of LTP in memory formation processes. Specifically, associativity denotes the amplification of connections when weak stimulus input is paired with a powerful one; synapse specificity posits that this potentiating effect only manifests on synaptic locations exhibiting coincidental activity within postsynaptic neurons, while cooperativity suggests stimulated neuron needs to attain an adequate threshold of depolarization before LTP can be induced again (Orsini and Maren, 2012).

There is support for the idea that memories are encoded by modification of synaptic strengths through cellular mechanisms such as LTP and LTD (Nabavi et al., 2014). The paper by Nabavi et al. (2014) shows that fear conditioning, a type of associative memory, can be inactivated and reactivated by LTD and LTP, respectively. The findings of the paper support a causal link between these synaptic processes and memory. Moreover, the paper suggests that LTP is used to form neuronal assemblies that represent a memory, and LTD could be used to disassemble them and thereby inactivate a memory (Nabavi et al., 2014). Hippocampal LTD has been found to play an essential function in regulating synaptic strength and forming memories, such as long-term spatial memory (Ge et al., 2010). However, it is vital to bear in mind that studies carried out on LTP exceed those done on LTD; hence the literature on it needs to be more extensive (Malenka and Bear, 2004; Nabavi et al., 2014).

## Cellular consolidation and memory

For an event to be remembered, it must form physical connections between neurons in the brain, which creates a “memory trace.” This memory trace can then be stored as long-term memory (Langille and Brown, 2018). The formation of a memory engram is an intricate process requiring neuronal depolarization and the influx of intracellular calcium (Mank and Griesbeck, 2008; Josselyn et al., 2015; Xu et al., 2017). This initiation leads to a cascade involving protein transcription, structural and functional changes in neural networks, and stabilization during the quiescence period, followed by complete consolidation for its success. Interference from new learning events or disruption caused due to inhibition can abort this cycle leading to incomplete consolidation (Josselyn et al., 2015).

Cyclic-AMP response element binding protein (CREB) has been identified as an essential transcription factor for memory formation (Orsini and Maren, 2012). It regulates the expression of PRPs and enhances neuronal excitability and plasticity, resulting in changes to the structure of cells, including the growth of dendritic spines and new synaptic connections. Blockage or enhancement of CREB in certain areas can affect subsequent consolidation at a systems level–decreasing it prevents this from occurring, while aiding its presence allows even weak learning conditions to produce successful memory formation (Orsini and Maren, 2012; Kandel et al., 2014).

Strengthening weakly encoded memories through the synaptic tagging and capture hypothesis may play an essential role in cellular consolidation. Retroactive memory enhancement has also been demonstrated in human studies, mainly when items are initially encoded with low strength but later paired with shock after consolidation (Dunsmoor et al., 2015). The synaptic tagging and capture theory (STC) and its extension, the behavioral tagging hypothesis (BT), have both been used to explain synaptic specificity and the persistence of plasticity (Moncada et al., 2015). STC proposed that electrophysiological activity can induce long-term changes in synapses, while BT postulates similar effects of behaviorally relevant neuronal events on learning and memory models. This hypothesis proposes that memory consolidation relies on combining two distinct processes: setting a “learning tag” and synthesizing plasticity-related proteins (*De novo* protein synthesis, increased CREB levels, and substantial inputs to nearby synapses) at those tagged sites. BT explains how it is possible for event episodes with low-strength inputs or engagements can be converted into lasting memories (Lynch, 2004; Moncada et al., 2015). Similarly, the emotional tagging hypothesis posits that the activation of the amygdala in emotionally arousing events helps to mark experiences as necessary, thus enhancing synaptic plasticity and facilitating transformation from transient into more permanent forms for encoding long-term memories (Richter-Levin and Akirav, 2003; Zhu et al., 2022).

Cellular consolidation, the protein synthesis-dependent processes observed in rodents that may underlie memory formation and stabilization, has been challenging to characterize in humans due to the limited ability to study it directly (Bermudez-Rattoni, 2010). Additionally, multi-trial learning protocols commonly used within human tests as opposed to single-trial experiments conducted with non-human subjects suggest there could be interference from subsequent information that impedes individual memories from being consolidated reliably. This raises important questions regarding how individuals can still form strong and long-lasting memories when exposed to frequent stimuli outside controlled laboratory conditions. Although this phenomenon remains undiscovered by science, it is of utmost significance for gaining a deeper understanding of our neural capacities (Genzel and Wixted, 2017).

The establishment of distributed memory traces requires a narrow temporal window following the initial encoding process, during which cellular consolidation occurs (Nader and Hardt, 2009). Once this period ends and consolidation has been completed, further protein synthesis inhibition or pharmacological disruption will be less effective at altering pre-existing memories and interfering with new learning due to the stabilization of the trace in its new neuronal network connections (Nader and Hardt, 2009). Thus, systems consolidation appears critical for the long-term maintenance of memory within broader brain networks over extended periods after their formation (Bermudez-Rattoni, 2010).

## System consolidation and memory

Information is initially stored in both the hippocampus and neocortex (Dudai et al., 2015). The hippocampus subsequently guides a gradual process of reorganization and stabilization whereby information present within the neocortex becomes autonomous from that in the hippocampal store. Scholars have termed this phenomenon “standard memory consolidation model” or “system consolidation” (Squire et al., 2015).

The Standard Model suggests that information acquired during learning is simultaneously stored in both the hippocampus and multiple cortical modules. Subsequently, it posits that over a period of time which may range from weeks to months or longer, the hippocampal formation directs an integration process by which these various elements become enclosed into single unified structures within the cortex (Gilboa and Moscovitch, 2021; Howard et al., 2022). These newly learned memories are then assimilated into existing networks without interference or compression when necessary (Frankland and Bontempi, 2005). It is important to note that memory engrams already exist within cortical networks during encoding. They only need strengthening through links enabled by hippocampal assistance-overtime allowing remote memory storage without reliance on the latter structure. Data appears consistent across studies indicating that both AMPA-and NMDA receptor-dependent “tagging” processes occurring within the cortex are essential components of progressive rewiring, thus enabling longer-term retention (Takeuchi et al., 2014; Takehara-Nishiuchi, 2020).

Recent studies have additionally demonstrated that the rate of system consolidation depends on an individual’s ability to relate new information to existing networks made up of connected neurons, popularly known as “schemas” (Robin and Moscovitch, 2017). In situations where prior knowledge is present and cortical modules are already connected at the outset of learning, it has been observed that a hippocampal-neocortical binding process occurs similarly to when forming new memories (Schlichting and Preston, 2015). The proposed framework involves the medial temporal lobe (MTL), which is involved in acquiring new information and binds different aspects of an experience into a single memory trace. In contrast, the medial prefrontal cortex (mPFC) integrates this information with the existing knowledge (Zeithamova and Preston, 2010; van Kesteren et al., 2012). During consolidation and retrieval, MTL is involved in replaying memories to the neocortex, where they are gradually integrated with existing knowledge and schemas and help retrieve memory traces. During retrieval, the mPFC is thought to use existing knowledge and schemas to guide retrieval and interpretation of memory. This may involve the assimilation of newly acquired information into existing cognitive schemata as opposed to the comparatively slow progression of creating intercortical connections (Zeithamova and Preston, 2010; van Kesteren et al., 2012, 2016).

Medial temporal lobe structures are essential for acquiring new information and necessary for autobiographical (episodic) memory (Brown et al., 2018). The consolidation of autobiographical memories depends on a distributed network of cortical regions. Brain areas such as entorhinal, perirhinal, and parahippocampal cortices are essential for learning new information; however, they have little impact on the recollection of the past (Squire et al., 2015). The hippocampus is a region of the brain that forms episodic memories by linking multiple events to create meaningful experiences (Cooper and Ritchey, 2019). It receives information from all areas of the association cortex and cingulate cortex, subcortical regions via the fornix, as well as signals originating within its entorhinal cortex (EC) and amygdala regarding emotionally laden or potentially hazardous stimuli (Sorensen, 2009). Such widespread connectivity facilitates the construction of an accurate narrative underpinning each remembered episode, transforming short-term into long-term recollections (Richter-Levin and Akirav, 2003).

Researchers have yet to establish a consensus regarding where semantic memory information is localized within the brain (Roldan-Valadez et al., 2012). Some proponents contend that such knowledge is lodged within perceptual and motor systems, triggered when we initially associate with a given object. This point of view is supported by studies highlighting how neural activity occurs initially in the occipital cortex, followed by left temporal lobe involvement during processing and pertinent contributions to word selection/retrieval via activation of left inferior frontal cortices (Patterson et al., 2007). Moreover, research indicates elevated levels of fusiform gyrus engagement (a ventral surface region encompassing both temporal lobes) occurring concomitantly with verbal comprehension initiatives, including reading and naming tasks (Patterson et al., 2007).

Research suggests that the hippocampus is needed for a few years after learning to support semantic memory (factual information), yet, it is not needed for the long term (Squire et al., 2015). However, some forms of memory remain dependent on the hippocampus, such as the retrieval of spatial memory (Wiltgen et al., 2010). Similarly, the Multiple-trace theory (Moscovitch et al., 2006), also known as the transformation hypothesis (Winocur and Moscovitch, 2011), posits that hippocampal engagement is necessary for memories that retain contextual detail such as episodic memories. Consolidation of memories into the neocortex is theorized to involve a loss of specific finer details, such as temporal and spatial information, in addition to contextual elements. This transition ultimately results in an evolution from episodic memory toward semantic memory, which consists mainly of gist-based facts (Moscovitch et al., 2006).

## Sleep and memory consolidation

Sleep is an essential physiological process crucial to memory consolidation (Siegel, 2001). Sleep is divided into two stages: Non-rapid Eye Movement (NREM) sleep and Rapid Eye Movement (REM) sleep. NREM sleep is divided into three stages: N1, N2, and N3 (AKA Slow Wave Sleep or SWS) (Rasch and Born, 2013). Each stage displays unique oscillatory patterns and phenomena responsible for consolidating memories in distinct ways. The first stage, or N1 sleep, is when an individual transitions between wakefulness and sleep. This type of sleep is characterized by low-amplitude, mixed-frequency brain activity. N1 sleep is responsible for the initial encoding of memories (Rasch and Born, 2013). The second stage, or N2 sleep, is characterized by the occurrence of distinct sleep spindles and K-complexes in EEG. N2 is responsible for the consolidation of declarative memories (Marshall and Born, 2007). The third stage of sleep N3, also known as slow wave sleep (SWS), is characterized by low-frequency brain activity, slow oscillations, and high amplitude. The slow oscillations which define the deepest stage of sleep are trademark rhythms of NREM sleep. These slow oscillations are delta waves combined to indicate slow wave activity (SWA), which is implicated in memory consolidation (Tononi and Cirelli, 2003; Stickgold, 2005; Kim et al., 2019). Sleep spindles are another trademark defining NREM sleep (Stickgold, 2005). Ripples are high-frequency bursts, and when combined with irregularly occurring sharp waves (high amplitude), they form the sharp-wave ripple (SWR). These spindles and the SWRs coordinate the reactivation and redistribution of hippocampus-dependent memories to neocortical sites (Ngo et al., 2020; Girardeau and Lopes-dos-Santos, 2021). The third stage is also responsible for the consolidation of procedural memories, such as habits and motor skills (Diekelmann and Born, 2010). During SWS, there is minimal cholinergic activity and intermediate noradrenergic activity (Datta and MacLean, 2007).

Finally, the fourth stage of sleep is REM sleep, characterized by phasic REMs and muscle atonia (Reyes-Resina et al., 2021). During REM sleep, there is high cholinergic activity, serotonergic and noradrenergic activity are at a minimum, and high theta activity (Datta and MacLean, 2007). REM sleep is also characterized by local increases in plasticity-related immediate-early gene activity, which might favor the subsequent synaptic consolidation of memories in the cortex (Ribeiro, 2007; Diekelmann and Born, 2010; Reyes-Resina et al., 2021). The fourth stage of sleep is responsible for the consolidation of emotional memories and the integration of newly acquired memories into existing knowledge structures (Rasch and Born, 2013). Studies indicate that the cholinergic system plays an imperative role in modifying these processes by toggling the entire thalamo-cortico-hippocampal network between distinct modes, namely high Ach encoding mode during active wakefulness and REM sleep and low Ach consolidation mode during quiet wakefulness and NREM sleep (Bergmann and Staresina, 2017; Li et al., 2020). Consequently, improving neocortical hippocampal communication results in efficient memory encoding/synaptic plasticity, whereas hippocampo-neocortical interactions favor better systemic memory consolidation (Diekelmann and Born, 2010).

The dual process hypothesis of memory consolidation posits that SWS facilitates declarative, hippocampus-dependent memory, whereas REM sleep facilitates non-declarative hippocampus-independent memory (Maquet, 2001; Diekelmann and Born, 2010). On the other hand, the sequential hypothesis states that different sleep stages play a sequential role in memory consolidation. Memories are encoded during wakefulness, consolidated during NREM sleep, and further processed and integrated during REM sleep (Rasch and Born, 2013). However, there is evidence present that contradicts the sequential hypothesis. A study by Goerke et al. (2013) found that declarative memories can be consolidated during REM sleep, suggesting that the relationship between sleep stages and memory consolidation is much more complex than a sequential model. Moreover, other studies indicate the importance of coordinating specific sleep phases with learning moments for optimal memory retention. This indicates that the timing of sleep has more influence than the specific sleep stages (Gais et al., 2006). The active system consolidation theory suggests that an active consolidation process results from the selective reactivation of memories during sleep; the brain selectively reactivates newly encoded memories during sleep, which enhances and integrates them into the network of pre-existing long-term memories (Born et al., 2006; Howard et al., 2022). Research has suggested that slow-wave sleep (SWS) and rapid eye movement (REM) sleep have complementary roles in memory consolidation. Declarative and non-declarative memories benefiting differently depending on which sleep stage they rely on (Bergmann and Staresina, 2017). Specifically, during SWS, the brain actively reactivates and reorganizes hippocampo-neocortical memory traces as part of system consolidation. Following this, REM sleep is crucial for stabilizing these reactivated memory traces through synaptic consolidation. While SWS may initiate early plastic processes in hippocampo-neocortical memory traces by “tagging” relevant neocortico-neocortical synapses for later consolidation (Frey and Morris, 1998), long-term plasticity requires subsequent REM sleep (Rasch and Born, 2007, 2013).

The active system consolidation hypothesis is not the only mechanism proposed for memory consolidation during sleep. The synaptic homeostasis hypothesis proposes that sleep is necessary for restoring synaptic homeostasis, which is challenged by synaptic strengthening triggered by learning during wake and synaptogenesis during development (Tononi and Cirelli, 2014). The synaptic homeostasis hypothesis assumes consolidation is a by-product of the global synaptic downscaling during sleep (Puentes-Mestril and Aton, 2017). The two models are not mutually exclusive, and the hypothesized processes probably act in concert to optimize the memory function of sleep (Diekelmann and Born, 2010).

Non-rapid eye movement sleep plays an essential role in the systems consolidation of memories, with evidence showing that different oscillations are involved in this process (Düzel et al., 2010). With an oscillatory sequence initiated by a slow frontal cortex oscillation (0.5–1 Hz) traveling to the medial temporal lobe and followed by a sharp-wave ripple (SWR) in the hippocampus (100–200 Hz). Replay activity of memories can be measured during this oscillatory sequence across various regions, including the motor cortex and visual cortex (Ji and Wilson, 2006; Eichenlaub et al., 2020). Replay activity of memory refers to the phenomenon where the hippocampus replays previously experienced events during sharp wave ripples (SWRs) and theta oscillations (Zielinski et al., 2018). During SWRs, short, transient bursts of high-frequency oscillations occur in the hippocampus. During theta oscillations, hippocampal spikes are ordered according to the locations of their place fields during behavior. These sequential activities are thought to play a role in memory consolidation and retrieval (Zielinski et al., 2018). The paper by Zielinski et al. (2018) suggests that coordinated hippocampal-prefrontal representations during replay and theta sequences play complementary and overlapping roles at different stages in learning, supporting memory encoding and retrieval, deliberative decision-making, planning, and guiding future actions.

Additionally, the high-frequency oscillations of SWR reactivate groups of neurons attributed to spatial information encoding to align synchronized activity across an array of neural structures, which results in distributed memory creation (Swanson et al., 2020; Girardeau and Lopes-dos-Santos, 2021). Parallel to this process is slow oscillation or slow-wave activity within cortical regions, which reflects synced neural firing and allows regulation of synaptic weights, which is in accordance with the synaptic homeostasis hypothesis (SHY). The SHY posits that downscaling synaptic strengths help incorporate new memories by avoiding saturation of resources during extended periods–features validated by discoveries where prolonged wakefulness boosts amplitude while it diminishes during stretches of enhanced sleep (Girardeau and Lopes-dos-Santos, 2021).

During REM sleep, the brain experiences “paradoxical” sleep due to the similarity in activity to wakefulness. This stage plays a significant role in memory processing. Theta oscillations which are dominant during REM sleep, are primarily observed in the hippocampus, and these are involved in memory consolidation (Landmann et al., 2014). There has been evidence of coherence between theta oscillations in the hippocampus, medial frontal cortex, and amygdala, which support their involvement in memory consolidation (Popa et al., 2010). During REM sleep, phasic events such as ponto-geniculo-occipital waves originating from the brainstem coordinate activity across various brain structures and may contribute to memory consolidation processes (Rasch and Born, 2013). Research has suggested that sleep-associated consolidation may be mediated by the degree of overlap between new and already known material whereby, if the acquired information is similar to the information one has learned, it is more easily consolidated during sleep (Tamminen et al., 2010; Sobczak, 2017).

In conclusion, understanding more about how the brains cycle through different stages of sleep, including specific wave patterns, offers valuable insight into the ability to store memories effectively. While NREM sleep is associated with SWRs and slow oscillations, facilitating memory consolidation and synaptic downscaling, REM sleep, characterized by theta oscillations and phasic events, contributes to memory reconsolidation and the coordination of activity across brain regions. By exploring the interactions between sleep stages, oscillations, and memory processes, one may learn more about how sleep impacts brain function and cognition in greater detail.

## Conclusion

Century has passed since we addressed memory, and several notable findings have moved from bench-to-bedside research. Several cross-talks between multidiscipline have been encouraged. Nevertheless, further research is needed into neurobiological mechanisms of non-declarative memory, such as conditioning (Gallistel and Balsam, 2014). Modern research indicates that structural change that encodes information is likely at the level of the synapse, and the computational mechanisms are implemented at the level of neural circuitry. However, it also suggests that intracellular mechanisms realized at the molecular level, such as micro RNAs, should not be discounted as potential mechanisms. However, further research is needed to study the molecular and structural changes brought on by implicit memory (Gallistel and Balsam, 2014).

The contribution of non-human animal studies toward our understanding of memory processes cannot be understated; hence recognizing their value is vital for moving forward. While this paper predominantly focused on cognitive neuroscience perspectives, some articles cited within this paper were sourced from non-human animal studies providing fundamental groundwork and identification of critical mechanisms relevant to human memories. A need persists for further investigation—primarily with humans—which can validate existing findings from non-human animals. Moving forward, it is prudent for researchers to bridge the gap between animal and human investigations done while exploring parallels and exploring unique aspects of human memory processes. By integrating findings from both domains, one can gain a more comprehensive understanding of the complexities of memory and its underlying neural mechanisms. Such investigations will broaden the horizon of our memory process and answer the complex nature of memory storage.

This paper attempted to provide an overview and summarize memory and its processes. The paper focused on bringing the cognitive neuroscience perspective on memory and its processes. This may provide the readers with the understanding, limitations, and research perspectives of memory mechanisms.

## Data availability statement

The original contributions presented in this study are included in the article/supplementary material, further inquiries can be directed to the corresponding author.

## Author contributions

SS and MKA: conceptualization, framework, and manuscript writing. AK: review and editing of the manuscript. All authors contributed to the article and approved the submitted version.
